# The antioxidant *N*-acetylcysteine promotes immune response and inhibits epithelial-mesenchymal transition to alleviate pulmonary fibrosis in chronic obstructive pulmonary disease by suppressing the VWF/p38 MAPK axis

**DOI:** 10.1186/s10020-021-00342-y

**Published:** 2021-09-03

**Authors:** Lanlan Zhu, Fei Xu, Xiuhua Kang, Jing Zhou, Qinqin Yao, Yang Lin, Wei Zhang

**Affiliations:** Department of Respiratory and Critical Care Medicine, The First Affiliated Hospital of Nanchang University, No. 17, Yongwaizheng Street, Nanchang, 330000 Jiangxi People’s Republic of China

**Keywords:** *N*-Acetylcysteine, Chronic obstructive pulmonary disease, Pulmonary fibrosis, Epithelial-mesenchymal transition, Immune response, VWF, p38 MAPK

## Abstract

**Background/aim:**

*N*-Acetylcysteine (NAC) demonstrates applications in the prevention of exacerbation of chronic obstructive pulmonary disease (COPD). COPD is often characterized by fibrosis of the small airways. This study aims at investigating the physiological mechanisms by which NAC might mediate the pulmonary fibrosis in COPD.

**Methods:**

A total of 10 non-smokers without COPD and 10 smokers with COPD were recruited in this study, and COPD rat models were established. Cigarette smoke extract (CSE) cell models were constructed. The gain- or loss-of-function experiments were adopted to determine the expression of VWF and the extent of p38 MAPK phosphorylation, levels of interleukin-6 (IL-6), tumor necrosis factor-α (TNF-α), and immunoglobulins (IgG, IgM and IgA) in the serum of COPD rats and supernatant of alveolar epithelial cells and to detect cell invasion and migration and the ratio of CD3^+^, CD4^+^, CD8^+^ and CD4^+^/CD8^+^T lymphocytes.

**Results:**

Expression of VWF and the extent of p38 MAPK phosphorylation were increased in COPD. NAC inhibited p38 MAPK phosphorylation by reducing the VWF expression. NAC could inhibit cell migration and invasion, elevate E-cadherin expression, the ratio of CD3^+^, CD4^+^, CD8^+^ and CD4^+^/CD8^+^T lymphocytes, and levels of IgG, IgA, and IgM, and reduce N-cadherin expression and levels of IL-6 and TNF-α in CSE cells and serum of COPD rats. NAC promoted immune response and suppressed epithelial-mesenchymal transformation (EMT) to relieve COPD-induced pulmonary fibrosis in vitro and in vivo by inhibiting the VWF/p38 MAPK axis.

**Conclusions:**

Collectively, NAC could ameliorate COPD-induced pulmonary fibrosis by promoting immune response and inhibiting EMT process via the VWF/p38 MAPK axis, therefore providing us with a potential therapeutic target for treating COPD.

**Supplementary Information:**

The online version contains supplementary material available at 10.1186/s10020-021-00342-y.

## Background

Chronic obstructive pulmonary disease (COPD) is a smoking-related disease with a high incidence and high mortality in the world (Xia et al. 2019; Yu et al. 2019). COPD is characterized by inflammation and fibrosis of the small airways and destruction of lung parenchyma (emphysema), which eventually leads to irreversible airflow obstruction (Li et al. 2013; Barnes 2020). Inflammatory response induced by cigarette smoke (CS) in the development of COPD involves innate immunity and adaptive immunity, which is mediated by a complex network of immune cell types, molecular mediators and lung tissue (Pan et al. 2016). Small airway epithelial epithelial-mesenchymal transformation (EMT) is the main cause of COPD small airway wall thickening and fibrosis (Sohal 2017). In recent years, there are accumulating researches on COPD, the specific mechanisms of the pathogenesis of COPD remains to be elucidated, and there is still no effective prevention and treatment (Negewo et al. 2015; Zhu et al. 2018; Mandru et al. 2021; Poole et al. 2019).

*N*-Acetylcysteine (NAC), the acetylated variant of the amino acid L-cysteine, has been widely used as the specific antidote for acetaminophen overdose and proved to participate in treating COPD as a mucolytic agent (Li et al. 2013; Millea 2009; Calverley et al. 2021). Prior studies have explored that NAC is beneficial in some pulmonary diseases such as idiopathic pulmonary fibrosis and cystic fibrosis (Shen et al. 2014; Burns et al. 2019). NAC possesses well-described antioxidant, anti-inflammatory, and mucolytic properties, making it attractive as a potential COPD therapy (Johnson et al. 2016). Moreover, NAC could reduce the expression of von Willebrand factor (VWF) at intramolecular disulfide bonds (Shortt et al. 2014). VWF exerts great effects on hemostasis, acting directly to recruit platelets to sites of vascular damage, and indirectly by chaperoning clotting factor VIII to reduce its degradation and clearance (Nightingale and Cutler 2013). The roles of VWF in endothelial dysfunction and inflammation in COPD have been demonstrated (Polosa et al. 2013). In addition, NAC could also inhibit the activation of p38 mitogen-activated protein kinase (MAPK) to reduce oxidative stress and to diminish apoptosis (Gong et al. 2016). The p38 MAPK family of serine/threonine protein kinases comprises four isoforms (p38α, p38β, p38γ, and p38δ) that are activated by inflammatory stimuli (Khorasani et al. 2015). p38 MAPK cascade is implicated in the inflammatory response of multiple chronic inflammatory diseases, including COPD (Elkhawad et al. 2012). However, the mechanism by which NAC-mediated VWF/p38 MAPK affects pulmonary fibrosis in COPD involving the immune response and EMT is still poorly understood, highlighting a major gap in knowledge given that NAC-mediated VWF/p38 MAPK may be of significance to pulmonary fibrosis in COPD. Hence, we hypothesized that the transfer of NAC might relieve pulmonary fibrosis in COPD by regulating the VWF/p38 MAPK axis.

## Materials and methods

### Ethics statement

All participants signed informed consent, and this study was performed with the approval of the Ethics Committee of the First Affiliated Hospital of Nanchang University. Animal experiments were approved by the Ethics Review Committee of the First Affiliated Hospital of Nanchang University and conducted according to the guidelines of the Care and Use of Laboratory Animals by the National Institute of Health, China.

### Network pharmacology analysis

The keyword "*N*-acetylcysteine” was searched using the drug database PubChem (https://pubchem.ncbi.nlm.nih.gov/) and DGIdb database (http://dgidb.org/) to predict drug target gene interactions. The Cytoscape software was utilized to perform network pharmacology visualization processing of drug target genes. The “clusterProfiler” package of the R language was utilized to conduct enrichment analysis of drug target genes (http://www.bioconductor.org/packages/release/bioc/html/clusterProfiler.html). The keyword “Chronic Obstructive Pulmonary Disease” was retrieved using the ctd database (http://ctdbase.org/) and GeneCards database (https://www.genecards.org/) to predict COPD-related genes, and the intersected disease genes were obtained using the jvenn tool (http://jvenn.toulouse.inra.fr/app/example.html). The STRING database (https://string-db.org/) was employed to retrieve a candidate gene with the Homo sapiens as defined species. Protein–protein interaction (PPI) network of the target construct disease genes was constructed to obtain the genes with interacted relationship. The Cytoscape software was utilized for pharmacological network visualization of interacted genes. The Node number and Edge number of PPI network were calculated using R language.

The intersection of the pharmaceutical-target disease genes was obtained using by jvenn. The GEO analysis database (https://www.ncbi.nlm.nih.gov/geo/) was used to search keywords “COPD” to search the disease-related chips, and 6 groups of lung tissues of COPD patients who smoke and 5 groups of lung tissues of COPD patients who do not smoke. The “limma” package of the R language (http://www.bioconductor.org/packages/release/bioc/html/limma.html) was used to perform differential analysis of COPD related genes with |logFC|> 1 and the *p* < 0.05 as the criteria. The Metascape website (https://metascape.org/gp/index.html #/main/step1) was employed for enrichment analysis of disease target genes.

### Clinical samples

Twenty subjects were recruited for this study, including 10 non-smokers without COPD, 10 smokers with COPD. The clinical and pulmonary function characteristics are shown in Additional file 1: Table S1. The inclusion criteria were as follows: All subjects underwent pulmonary nodule resection. A diagnosis of COPD was made according to the guidelines of the Global Initiative for Chronic Obstructive Lung Disease. COPD patients in our study were defined as having a phenotype of chronic bronchitis and had a history of exposure to cigarette smoke. All patients took a pulmonary function test, and forced expiratory volume in 1 s (FEV1)/forced vital capacity (FVC) < 0.70 was used to confirm the airflow limitation to diagnose COPD. All patients were subjected to serum protein electrophoresis tests, and there were no subjects with alpha-1 antitrypsin deficiency. Patients had no history of exposure to occupational dust or chemicals or indoor or outdoor air pollution. The exclusion criteria were as follows: Patients with comorbidities, including interstitial lung disease, heart failure, asthma, and neuromuscular disease, were excluded. None of the subjects had suffered from any respiratory tract infection or received any glucocorticoids or antibiotics during the month preceding the study. All lung samples were obtained from the First Affiliated Hospital of Nanchang University (Nanchang, China). For each specimen, two tissue blocks (sample size 15–25 mm) were taken from the sub-pleural parenchyma of the lobe obtained in surgery, and they were from at least 5 cm away from the margin of the diseased regions.

### Establishment of COPD rat models

Except for the normal group, all rats were anesthetized with 100 mg/kg sodium pentobarbital (P3761, Sigma-Aldrich Chemical Company, St Louis, MO, USA), and the trachea were exposed to 200 μL of lipopolysaccharide (LPS, L2630, Sigma-Aldrich) at a concentration of 1 mg/mL). Subsequently, the rats were placed with a 50 g sawdust and 0.682 g of cigarettes (tar 13.5 mg/G, nicotine 0.48 mg/g) mixed combustion smoke chamber. The rats were exposed to smoke, 30 min per day, for 28 days to establish a rat model of COPD. Rats were exposed to smoke while transtracheal injection adenovirus (10^8^ pfu/500 μL), once a week (Xia et al. 2019). At 2 days before COPD establishment (Rubio et al. 2004), the NAC treated group rats fed gastric the NAC (800 mg/kg, once a day) until the end of model establishment, and the control mice were perfused with PBS (Cai et al. 2009). Subsequently, the respiratory rate and body weight were monitored in rats. In addition, the tail vein blood (5 mL) was collected. The serum was centrifuged at 2000 rpm at 4 °C for 15 min and stored at − 80 °C for cytokine analysis (Wang et al. 2017).

COPD rats were treated with PBS and NAC, or transduced with adenovirus carrying overexpression (oe)-VWF, and/or short hairpin RNA (sh)-p38 MAPK as well as their corresponding negative control (NC) (oe-NC and sh-NC). The adenovirus was purchased from Shanghai Genechem Co., Ltd. (Shanghai, China). The silenced adenovirus was constructed using the GV119 vector, and the overexpression adenovirus was constructed using the GV314 vector. The primer sequence, vector construction, virus packaging and purification were completed by Genechem. The experiments were conducted according to the instructions.

### Evaluation of pulmonary function

On the 30th day after the drug treatment was initiated, respiratory rate and the peak expiratory flow (PEF) were monitored using a pulmonary function test apparatus for small animals (PFT, BUXCO, USA). The forced expiratory flow in 0.3 s (FEV_0.3_) was calculated by the forced vital capacity (FVC), and then the pulmonary function was assessed on the basis of the formula: FEV_0.3_/FVC × 100%.

### Cell isolation and culture

Sprague–Dawley (SD) rats (6 weeks) were euthanized by exsanguination under deep anesthesia (pentobarbital 100 mg/kg), and the lungs were removed. The lungs were then perfused with a perfusion solution (2.65 mM phosphate buffer pH 7.4, 135 mM NaCl, 4.86 mM glucose, 1.9 mM CaCl_2_, 5.3 mM KCl, 1.3 mM MgSO_4_, and 10 mM N-2-hydroxyethyl-piperazine-N'-2-ethanesulfonic acid buffer) via the pulmonary artery until they appeared white. After that, the lungs were digested at 37 °C with elastase that was injected into trachea. Subsequently, the lung tissue was minced and filtered through 140- and 30-μm nylon mesh filters. The filtered cells were centrifuged, and the cell pellet was resuspended into Dulbecco's modified Eagle's medium (DMEM) (Gibco Company, Grand Island, NY, USA) and incubated at 37 °C for 1 h. The unattached cells were collected and seeded on 24-well plates at 2 × 10^6^ cells/well. Then, the alveolar epithelial cells were cultured in DMEM containing 10% fetal bovine serum (FBS, Gibco) at 37 °C with 5% CO_2_ in a humidified incubator.

### Cell grouping and treatment

Cigarette smoke extract (CSE) was prepared by bubbling the smoke from two cigarettes into 20 mL of serum-free DMEM, which was then filtered with a 0.2 μm filter to sterilize the mixture. An optical density of 0.65 (320 nm) was considered to represent 100% CSE and was diluted in serum-free DMEM to 2% CSE. With the exception of the NC group, the cells were stimulated with CSE in combination with LPS (0.1 µg/mL) for 24 h. After that, the media were removed, and cells received other treatments (Wang et al. 2017). NAC was added to culture medium 4 h before cells were exposed to CSE, and the NAC medium was replaced every 24 h thereafter. The cells were pretreated with NAC for 4 h, followed by 10% CSE exposure for 72 h. Meanwhile, the cells were added with the corresponding lentivirus to measure the expression of related index (Zhang et al. 2012).

Cell grouping was as follows: oe-NC, oe-VWF, sh-NC, sh-p38 MAPK-1, sh-p38 MAPK-2; Control, CSE, CSE + PBS, CSE + NAC, CSE + NAC + oe-NC, CSE + NAC + oe-VWF, NAC + oe-NC + sh-NC, NAC + oe-VWF + sh-NC, and NAC + oe-VWF + sh-p38 MAPK group. The core plasmid (PLKO.1) and auxiliary plasmids (RRE, REV, Vsvg) inserted into the target gene silencing sequence were used to package the silent lentivirus. The core plasmid (Fugw-GFP, Plx304) and auxiliary plasmids (RRE, REV, Vsvg) inserted into the target gene cDNA sequence were used to package the overexpressed lentivirus. The lentivirus was purchased from Shanghai Sangon Biotechnology Co., Ltd. (Shanghai, China) and the primer sequence and plasmid construction were completed by Sangon (Additional file 1: Table S2). All procedures were conducted according to the instructions.

### Histological study

The left lung was isolated, fixed intratracheally with 2 mL of 4% formaldehyde, washed with PBS, and immersed in the same fixative for at least 24 h. After formaldehyde-fixation and paraffin-embedding, the specimens were sectioned at 3–4 μm and processed for a standard hematoxylin–eosin (HE) staining and Masson’s trichrome stain. A semi-quantitative morphometric analysis of lung injury was determined by particular histological score, according to the following scale: 0, normal lung; 1, septal congestion; 2, epithelial thickening; 3, septal inflammatory infiltrates; 4, alveolar hemorrhage and/or hyaline membranes; 5, massive disruption of lung architecture. Additionally, three random microscope fields in which the bronchiole diameters were < 100 μm (shortest path/lumen diameter, ≥ 0.7) at a magnification of × 100 were observed to determine the changes of thickness and area of the tube wall. The wall area/total bronchiole area (MA%) and the wall thickness/bronchiole diameter (MT%) were then calculated. Also, a muscular artery diameter of 50 to 100 μm was selected to evaluate small vessel remodeling at a magnification of × 200.

### Isolation and quantification of RNA

TRIzol reagent (16096020, Thermo Fisher Scientific, New York, USA) was used to extract total RNA. For mRNA detection, reverse transcription kit (RR047A, Takara Bio Inc., Otsu, Shiga, Japan) was used to perform reverse transcription to obtain cDNA. Reverse transcription quantitative polymerase chain reaction (RT-qPCR) was performed according to the instructions of RT-qPCR kit (Q511-02, Vazyme Biotech, Nanjing China). PCR amplification was performed using the Bio-rad real-time quantitative PCR instrument (CFX96). Glyceraldehyde-3-phosphate dehydrogenase (GAPDH) was used as an internal reference of VWF and p38 MAPK. The primer sequence and plasmid construction were completed by Sangon (Additional file 1: Table S3). The 2^−ΔΔCt^ method was used to quantify the relative expression of target genes.

### Western blot analysis

Total protein was extracted from the cells and tissues in radio-immunoprecipitation assay (RIPA) lysis buffer containing phenylmethylsulphonyl fluoride (PMSF). The proteins from cell nuclei and plasma were extracted according to the instructions (P0028, Beyotime Institute of Biotechnology, Shanghai, China). The supernatant was taken to determine protein concentration using a bicinchoninic acid (BCA) protein assay kit (Beyotime). The concentration of protein was adjusted to 1 μg/μL. The sample volume of each tube was set at 100 μL, denatured at 100 °C for 10 min, and stored at − 80 °C for further experiments. The sample was extracted and separated by 8–12% sodium dodecyl sulfate–polyacrylamide gel electrophoresis (SDS-PAGE), electrotransferred onto a polyvinylidene fluoride (PVDF) membrane (1620177, Bio-Rad, Richmond, Cal., USA). The membrane was blocked in 5% skimmed milk or 5% bovine serum albumin (BSA) at room temperature for 1 h, and then incubated overnight at 4 °C with the following primary antibodies: rabbit anti-GAPDH (2118, 1: 5000, Cell Signaling Technology, Beverly, MA, USA), rabbit anti-VWF (AB7356, 1: 1000, Sigma-Aldrich), rabbit anti-Collagen I (NB600-408, 1: 1000, Novus Biologicals, Littleton, CO, USA), rabbit anti-α-SMA (55135-1-AP, 1: 1000, Proteintech Group Inc., IL, USA), rabbit anti-p-p38 MAPK (4511, 1: 1000, Cell Signaling Technology), rabbit anti-p38 MAPK (9212, 1: 1000, Cell Signaling Technology), rabbit anti-N-Cadherin (ab18203, 1: 1000, Abcam Inc., Cambridge, UK), and rabbit anti-E-cadherin (20874-1-AP, 1: 1000, Proteintech). The membrane was incubated with the secondary antibody goat anti-rabbit immunoglobulin G (IgG) antibody (1: 10,000, ab97051, Abcam) on a shaker at room temperature. The next day, the membrane was washed thrice with TBST 3 times (5 min per wash) and incubated with the secondary antibody HRP-labeled goat anti-rabbit IgG (ab6721, 1: 5000, Abcam) at room temperature for 1 h. Thereafter, the immunocomplexes on the membrane were visualized using enhanced chemiluminescence (ECL) reagent (1705062, Bio-Rad). The membrane was exposed to light using Image Quant LAS 4000C gel imager (GE Company, USA). The relative protein expression was expressed as the ratio of the gray value of protein to be tested to that of internal reference (GAPDH).

### Enzyme-linked immunosorbent assay (ELISA)

The reagent kits IL-6 (PI328, Beyotime), tumor necrosis factor-α (TNF-α) (PT516, Beyotime), IgG (ab189578, Abcam), IgM (ab157738, Abcam), and IgA (ab157735, Abcam) were used to detect the expression of related factors in the serum of rats or the supernatant of alveolar epithelial cells.

### Immunohistochemical staining

The paraffin-embedded lung tissues from COPD patients or rats were cut into sections, which were baked at 60 °C for 20 min. The sections were sequentially placed in xylene for 15 min, soaked in xylene for 15 min again after replacing, and rehydrated in anhydrous alcohol for 5 min. Next, anhydrous alcohol was replaced, followed by hydration for 5 min, and then hydrated in 70% and 95% alcohol for 10 min. The sections were added with 3% H_2_O_2_ and soaked for 10 min at room temperature to block the endogenous peroxidase. The sections were added with citric acid buffer, cooked in a microwave oven for 3 min, incubated with antigen retrieval solution at room temperature for 10 min, and washed with PBS three times. The sections were added with normal goat serum blocking solution (Sangon) at room temperature for 20 min, and incubated with following diluted primary antibodies, rabbit anti-α-SMA (5513 5–1-AP, 1: 300, Proteintech), rabbit anti-VWF (AB7356, 1: 300, Sigma-Aldrich), and rabbit anti-p-p38 MAPK (4511, 1: 300, Cell Signaling Technology) at 4 °C overnight. After washing with PBS three times, the sections were incubated with secondary antibody goat anti-rabbit IgG (ab6721, 1: 500, Abcam) for 30 min, and with streptavidin biotin peroxidase complex (SABC) (Vector Labs, Burlingame, CA, USA) in a 37 °C incubator for 30 min. The sections were developed with chromogenic agent A, B, and C using DAB kit (Sigma-Aldrich) for 6 min, stained with hematoxylin for 30 s, dehydrated in ascending series of alcohol (70%, 80%, 90%, and 95% ethanol and anhydrous in sequence) for 2 min, immersed twice in xylene for 5 min and sealed with neutral resin before observation and counting under an upright microscope (BX63, Olympus Optical Co., Ltd., Tokyo, Japan).

### Transwell assay

Transwell migration assay: the Transwell chamber was coated with 50 μL Matrigel (354234, BD Biosciences, Franklin Lakes, NJ, USA), and solidified at 37 °C for 30 min. The coated chamber was washed with FBS-free medium. The medium without FBS was used to dilute cells to 2.5 × 10^4^ cells/mL. The upper chamber was added with 100 μL cell suspension, and the lower chamber was added with 500 μL medium containing 10% FBS. After 24 h, the chamber was taken out. The cells in the upper chamber were removed by cotton swabs, and the cells were fixed at room temperature with 4% paraformaldehyde for 30 min. Subsequently, the cells were stained with 0.1% crystal violet for 30 min, and 5 fields under a microscope were randomly selected to take pictures and count the number of cells. The cell migration assay was conducted without Matrigel. The other steps were the same. The photograph was taken under an inverted microscope (IXplore Pro, Olympus).

### Flow cytometry

Cell suspensions were treated with ammonium chloride-potassium (ACK) buffer (150 mmol/l NH_4_ Cl, 10 mmol/l KHCO_3_ and 0.1 mmol/l disodium ethylenediamine tetraacetic acid) and then stained with the following conjugated antibodies: CD3-PE (554833, BD Biosciences), CD4-fluorescein isothiocyanate (FITC) (561834, BD Biosciences), and CD8α-PE (554857, BD Biosciences). T lymphocytes were analyzed using analyzed on a FACSCanto (BD Biosciences) running FACSDiva software (version 5.01).

### Statistical analysis

All data were presented as mean ± standard deviation. Data with normal distribution and homogeneity of variance between two groups were analyzed using independent sample *t* test. For data comparison among multiple groups, one-way analysis of variance (ANOVA) with Tukey post hoc test was used. Data at different time points were analyzed using two-way ANOVA with Bonferroni post hoc test. Values of *p* < 0.05 were considered significant. Statistical analysis was performed using SPSS 21.0 (IBM Corp, Armonk, NY, USA).

## Results

### NAC relieves pulmonary fibrosis caused by COPD

**Fig. 1:**
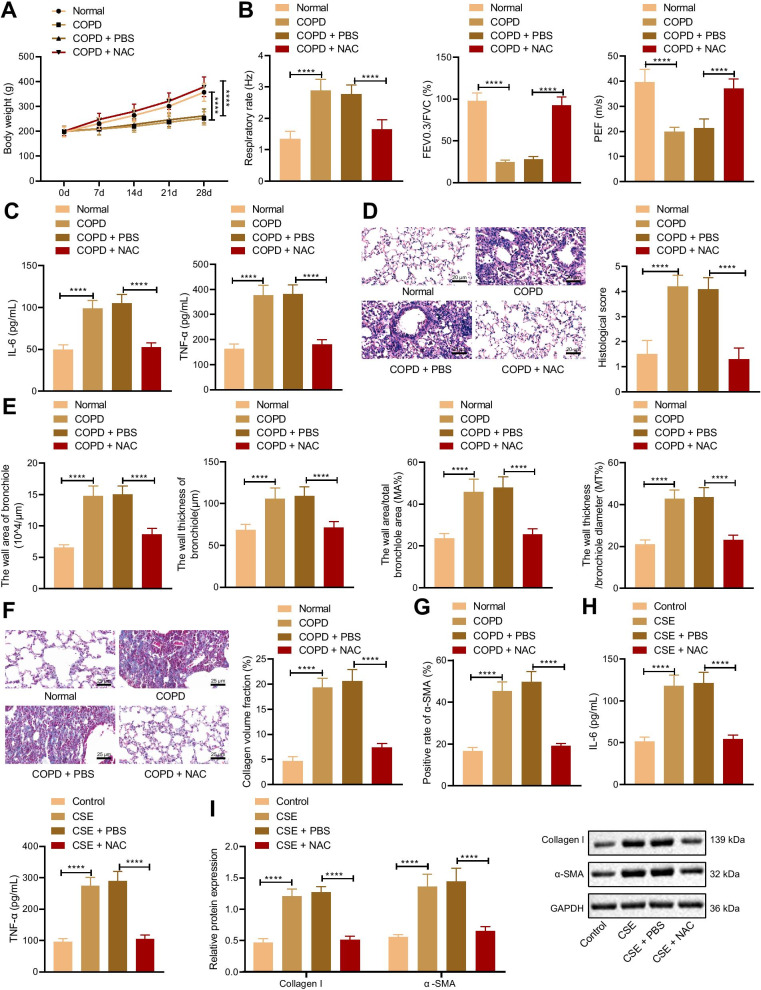
NAC could ameliorate the COPD-induced pulmonary fibrosis. COPD rats were treated with PBS or NAC (n = 10). **A** The weight of COPD rats. **B** Pulmonary function of COPD rats. **C** Levels of IL-6 and TNF-α in the serum of COPD rats measured by ELISA. **D** histological score of COPD rats detected by HE staining. **E** Bronchioles area, thickness bronchioles, the wall area/total bronchiole area (MA%) and the wall thickness/bronchiole diameter (MT%) of COPD rats; **F** Collagen volume fraction in lung tissues of COPD rats detected by Masson’s trichrome stain. **G** α-SMA level in lung tissues of COPD rats detected by Immunohistochemistry. Cells were treated with CSE and PBS or CSE and NAC. **H** Levels of IL-6 and TNF-α in cells measured by ELISA. **I** protein levels of Collagen I and α-SMA in cells determined by Western blot analysis. ****p < 0.0001. Data are shown as the mean ± standard deviation of three technical replicates. Data among multiple groups were compared by one-way ANOVA with Tukey’s post hoc test. Data at different time points were compared by two-way ANOVA with Bonferroni post hoc test

It has been reported that NAC can inhibit COPD development, while the mechanism is still unclear (Tse et al. 2014). In order to further verify the mechanism of NAC alleviating COPD, COPD rat models and CSE-induced cell models were constructed. The results of the weight detection of rats showed that compared with the normal rats, the weight of the COPD rats significantly reduced. Compared with the COPD rats treated with PBS, the weight of the COPD rats treated with NAC increased (Fig. 1A). Evaluation of pulmonary function presented that compared with normal rats, respiration rate of COPD rats elevated, and ratio of FEV_0.3_/FVC and PEF decreased. Compared with the COPD rats treated with PBS, COPD rats treated with NAC showed decreased respiration rate and elevated ratio of FEV_0.3_/FVC and PEF (Fig. 1B). ELISA displayed that, compared with the normal rats, levels of IL-6 and TNF-α elevated in COPD rats, which were decreased in COPD rats treated with NAC in comparison to COPD rats treated with PBS (Fig. 1C). HE staining exhibited that the COPD rats had larger alveolar spaces, and the increased number of infiltrated inflammatory cells and tissue scores compared with the normal rats. Compared with the COPD rats treated with PBS, COPD rats treated with NAC had less inflammatory cell infiltration, destruction of alveolar septum, and reduced tissue score (Fig. 1D). Compared normal rats, the wall area, the wall thickness of the bronchioles, the wall area/total bronchiole area (MA%) and the wall thickness/bronchiole diameter (MT%) in COPD rats increased. Compared with the COPD rats treated with PBS, the results were opposite in COPD rats treated with NAC (Fig. 1E). Masson’s trichrome stain and Immunohistochemistry presented that compared with normal rats, collagen volume fraction and α-SMA level increased, and compared with COPD rats treated with PBS, collagen volume fraction and α-SMA level reduced in COPD rats treated with NAC (Fig. 1F, G). The above results indicated that COPD rat models were successfully constructed, and NAC could alleviate pulmonary fibrosis in COPD rats.

The results of ELISA and Western blot analysis showed that compared with cells without treatment, levels of IL-6 and TNF-α and protein levels of Collagen I and α-SMA elevated in cells treated with CSE, while levels of IL-6 and TNF-α and protein levels of Collagen I and α-SMA decreased in cells treated with CSE and PBS compared with cells treated with CSE plus NAC (Fig. 1H, I). These results suggested that NAC was able to relieve pulmonary fibrosis caused by COPD.

### NAC inhibits the VWF expression

**Fig. 2:**
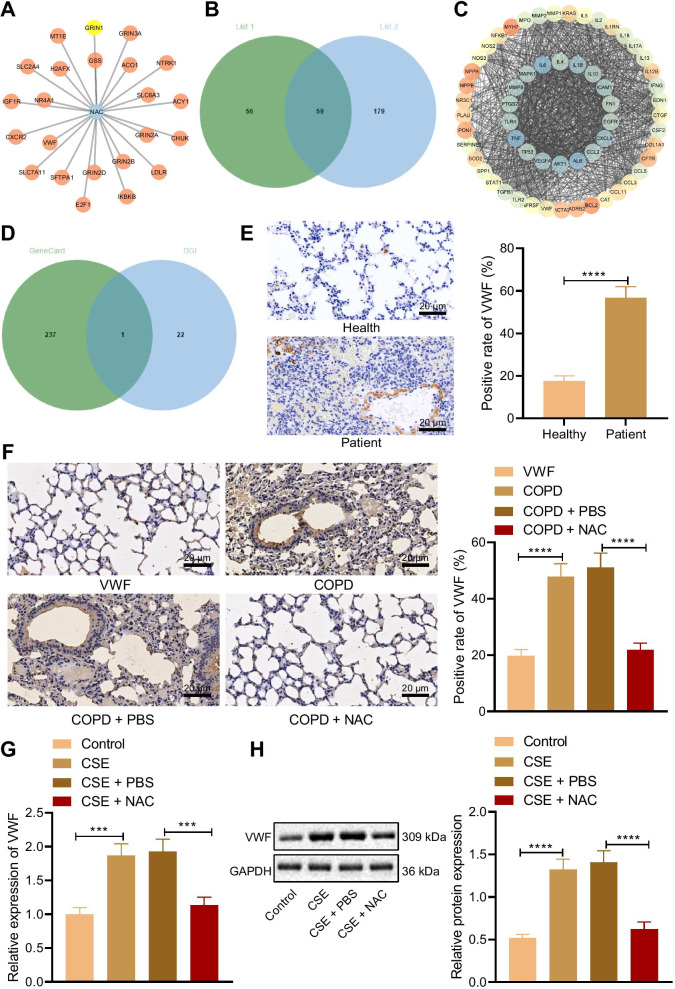
NAC suppresses the expression of VWF. **A** The interaction network of NAC and genes. **B** Venn map of intersection of COPD target genes. **C** PPI network of intersection of COPD target genes. **D** Venn map of intersection of NAC and COPD target genes. **E** VWF expression in lung tissues of healthy individuals and COPD patients measured by immunohistochemistry (n = 10). **F** VWF expression in lung tissues of COPD rats measured by immunohistochemistry (n = 10). **G** VWF mRNA level in cells determined using RT-qPCR. **H** VWF protein level in cells determined using Western blot analysis. ***p < 0.001. ****p < 0.0001. Data are shown as the mean ± standard deviation of three technical replicates. Data between two groups were compared by independent sample* t* test. Data among multiple groups were compared by one-way ANOVA with Tukey’s post hoc test

The network between NAC and its potential interaction genes were predicted through the drug database and the DGIdb database (Fig. 2A). Using network analysis, we obtained 59 target genes with intersection between the two databases (Fig. 2B). We constructed a PPI network of the 59 target genes through the String database to obtain the interaction relationship network of COPD target genes (Fig. 2C). Then the intersection of NAC and COPD target genes was detected, and one target gene, namely VWF, was obtained (Fig. 2D). Immunohistochemistry analysis results showed that compared with the healthy individuals, VWF expression increased in the lung tissues of COPD patients (Fig. 2E). Compared with the normal rats, VWF expression in the lung tissues of COPD rats significantly elevated, while VWF expression in the lung tissues of COPD rats treated with NAC decreased in comparison to COPD rats treated with PBS (Fig. 2F). In addition, RT-qPCR and Western blot analysis results exhibited that compared with cells without treatment, VWF expression in the cells treated with CSE increased, while compared with the cells treated with CSE and PBS, VWF expression reduced in the cells treated with CSE and NAC (Fig. 2G, H). These results confirmed that NAC reduced VWF expression.

### Overexpressed VWF weakens NAC therapeutic effect on COPD-induced pulmonary fibrosis

**Fig. 3:**
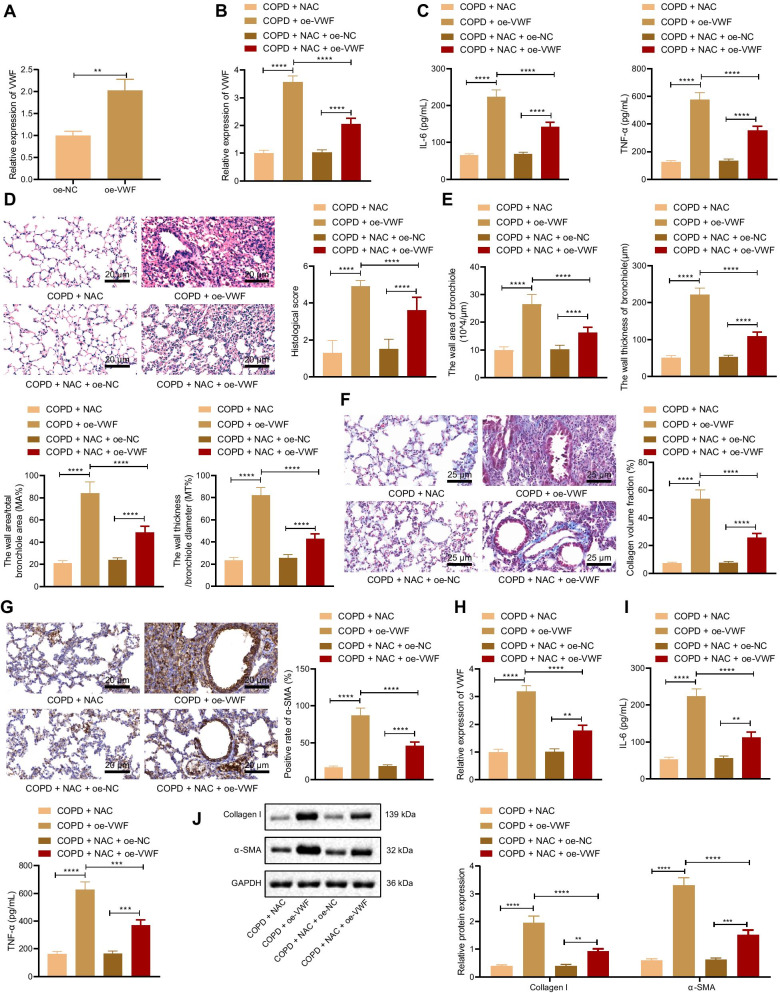
NAC relieves COPD-induced pulmonary fibrosis by inhibiting VWF. **A** Efficiency of VWF overexpression detected by RT-qPCR. COPD rats were treated with NAC and oe-VWF (with NAC and oe-NC as control) (n = 10). **B** VWF expression in lung tissues of COPD rats detected by RT-qPCR. **C** Levels of IL-6 and TNF-α in the serum of COPD rats measured by ELISA. **D** Histological score of COPD rats detected by HE staining. **E** Bronchioles area, thickness bronchioles, the wall area/total bronchiole area (MA%) and the wall thickness/bronchiole diameter (MT%) of COPD rats; **F** Collagen volume fraction in lung tissues of COPD rats detected by Masson’s trichrome stain. **G** α-SMA level in lung tissues of COPD rats detected by Immunohistochemistry. CSE cells were transduced NAC and oe-VWF (with NAC and oe-NC as control). **H** VWF expression in CSE cells detected by RT-qPCR. **I** Levels of IL-6 and TNF-α in CSE cells measured by ELISA. **J** Protein levels of Collagen I and α-SMA in CSE cells determined by Western blot analysis. **p < 0.05. ***p < 0.001. ****p < 0.0001. Data are shown as the mean ± standard deviation of three technical replicates. Data between two groups were compared by independent sample *t* test. Data among multiple groups were compared by one-way ANOVA with Tukey’s post hoc test

To further verify the therapeutic effect of overexpression of VWF to inhibit NAC on COPD-induced pulmonary fibrosis, RT-qPCR results displayed that VWF expression elevated in cells transduced with oe-VWF (Fig. 3A). The constructed oe-VWF and oe-NC vectors were injected into rats via tail vein, and 48 h later, relevant detection was conducted. RT-qPCR and ELISA data exhibited that compared with COPD rats treated with NAC and oe-NC, VWF expression and levels of IL-6 and TNF-α increased in lung tissues of COPD rats treated with NAC, oe-VWF, and oe-NAC. Relative to COPD rats treated with oe-VWF, COPD rats treated with NAC + oe-VWF showed much lower VWF expression and levels of IL-6 and TNF-α (Fig. 3B, C). HE staining analysis results exhibited that the COPD rats treated with NAC and oe-VWF had less inflammatory cell infiltration, less destruction of the alveolar septum, and complete columnar epithelial cells compared with those treated with NAC and oe-NC, while opposite trends were noted in the presence of NAC + oe-VWF compared with oe-VWF alone (Fig. 3D). Compared with COPD rats treated with NAC and oe-NC, the wall area, the wall thickness of the bronchioles, the wall area/total bronchiole area (MA%) and the wall thickness/bronchiole diameter (MT%) were all increased in lung tissues of COPD rats treated with NAC and oe-VWF. A pronounced decline was observed in the wall area, the wall thickness of the bronchioles, the wall area/total bronchiole area (MA%) and the wall thickness/bronchiole diameter (MT%) in lung tissues of COPD rats treated with NAC + oe-VWF compared with those treated with individual oe-VWF (Fig. 3E). Masson’s trichrome stain and Immunohistochemistry presented that compared with COPD rats treated with NAC and oe-NC, collagen volume fraction and α-SMA level increased in lung tissues of COPD rats treated with NAC and oe-VWF. In addition, collagen volume fraction and α-SMA level were much lower in lung tissues of COPD rats treated with NAC + oe-VWF than treatment with oe-VWF alone (Fig. 3F, G).

Furthermore, RT-qPCR, ELISA, and Western blot analysis showed that compared with CSE cells transduced with NAC and oe-NC, VWF expression, levels of IL-6 and TNF-α, and protein levels of Collagen I and α-SMA elevated in CSE cells transduced with NAC and oe-VWF. Additionally, treatment with NAC + oe-VWF led to decreased VWF expression, levels of IL-6 and TNF-α, and protein levels of Collagen I and α-SMA as compared to treatment with oe-VWF alone (Fig. 3H–J). It can be concluded that overexpressed VWF suppresses NAC therapeutic effect on COPD-induced pulmonary fibrosis.

At the same time, we injected the sh-VWF plasmid into the mouse COPD model via tail vein. Following knockdown of VWF, the expression of VWF was reduced (Additional file 2: Fig. S1A). In addition, HE staining data showed that the pathological degree of the sh-VWF-treated rats was weakened and NAC treatment failed to cause a significant effect on the reduced pathological degree induced by sh-VWF (Additional file 2: Fig. S1B, C). Combining the results of VWF overexpression, it showed that the therapeutic effect of NAC on COPD depended on VWF.

### NAC reduces p38 MAPK phosphorylation by inhibiting VWF

**Fig. 4:**
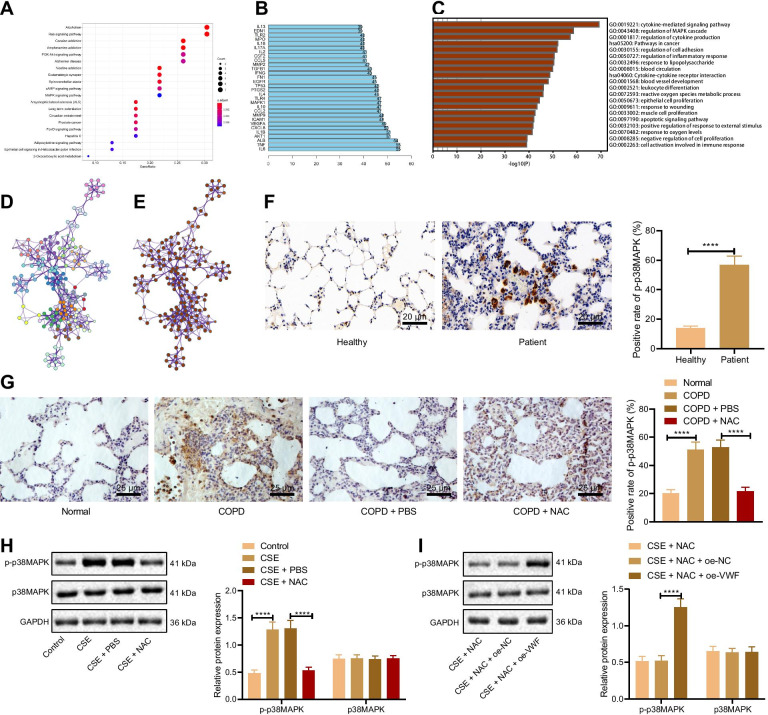
NAC inhibits VWF expression to reduces p38 MAPK phosphorylation. **A** KEGG enrichment analysis of NAC target genes. The abscissa represents the number of involved genes, and the ordinate represents the enrichment entry name. **B** the number of genes from PPI network interacted with genes. **C** GO enrichment analysis of COPD target genes. **D** GO enrichment analysis of biological pathways related to COPD target genes. **E** P value of GO enrichment analysis of biological pathways related to COPD target genes. **F** The extent of p38 MAPK phosphorylation in the lung tissues of healthy individuals and COPD patients measured by immunohistochemistry (n = 10). **G** The extent of p38 MAPK phosphorylation in lung tissues of COPD rats measured by immunohistochemistry (n = 10). **H** The extent of p38 MAPK phosphorylation and protein levels of and p38 MAPK in cells determined using Western blot analysis. CSE cells were transduced NAC and oe-VWF (with NAC and oe-NC as control). **I** The extent of p38 MAPK phosphorylation and protein levels of p38 MAPK in cells determined using Western blot analysis. ****p < 0.0001. Data are shown as the mean ± standard deviation of three technical replicates. Data between two groups were compared by independent sample *t* test. Data among multiple groups were compared by one-way ANOVA with Tukey’s post hoc test

The R language was performed for enrichment analysis of NAC to obtain MAPK signaling pathway (Fig. 4A). The higher degree genes from PPI network interacted with the genes were obtained. After differential analysis, MAPK was obtained and downregulated (GSE106986). MAPK signaling pathway was obtained through enrichment analysis of COPD target genes using Metascape website (Fig. 4C–E).

First, immunohistochemistry displayed that compared with the healthy individuals, the extent of p38 MAPK phosphorylation was elevated in lung tissues of COPD patients (Fig. 4F). Compared with normal rats, the extent of p38 MAPK phosphorylation was also elevated in the lung tissues of COPD rats. Conversely, compared with the COPD rats treated with PBS, the extent of p38 MAPK phosphorylation was decreased in the lung tissues of COPD rats treated with NAC (Fig. 4G). Western blot analysis data presented that compared with the cells without treatment, the extent of p38 MAPK phosphorylation was increased in cells treated with CSE, and compared to CSE cells treated with PBS, it was reduced in CSE cells treated with NAC (Fig. 4H). These results suggested that NAC reduces the extent of p38 MAPK phosphorylation. Furthermore, Western blot analysis exhibited that compared with the CSE cells transduced with NAC and oe-NC, CSE cells transduced with NAC and oe-VWF showed increased extent of p38 MAPK phosphorylation (Fig. 4I). The above-mentioned findings demonstrated that NAC can reduce the phosphorylation level of p38 MAPK by inhibiting VWF.

In order to further verify whether VWF overexpression or VWF knockout mediates the phosphorylation of p38 MAPK, we overexpressed VWF on isolated primary alveolar epithelial cells. The results of Western blot analysis showed that VWF induced the phosphorylation of p38 MAPK but an opposite result was noted in the absence of VWF (Additional file 3: Fig. S2A, B). These results indicated that VWF can regulate the phosphorylation of p38 MAPK, but the molecular mechanism warrant further investigation.

### NAC suppresses VWF/p38 MAPK axis to inhibit EMT

**Fig. 5:**
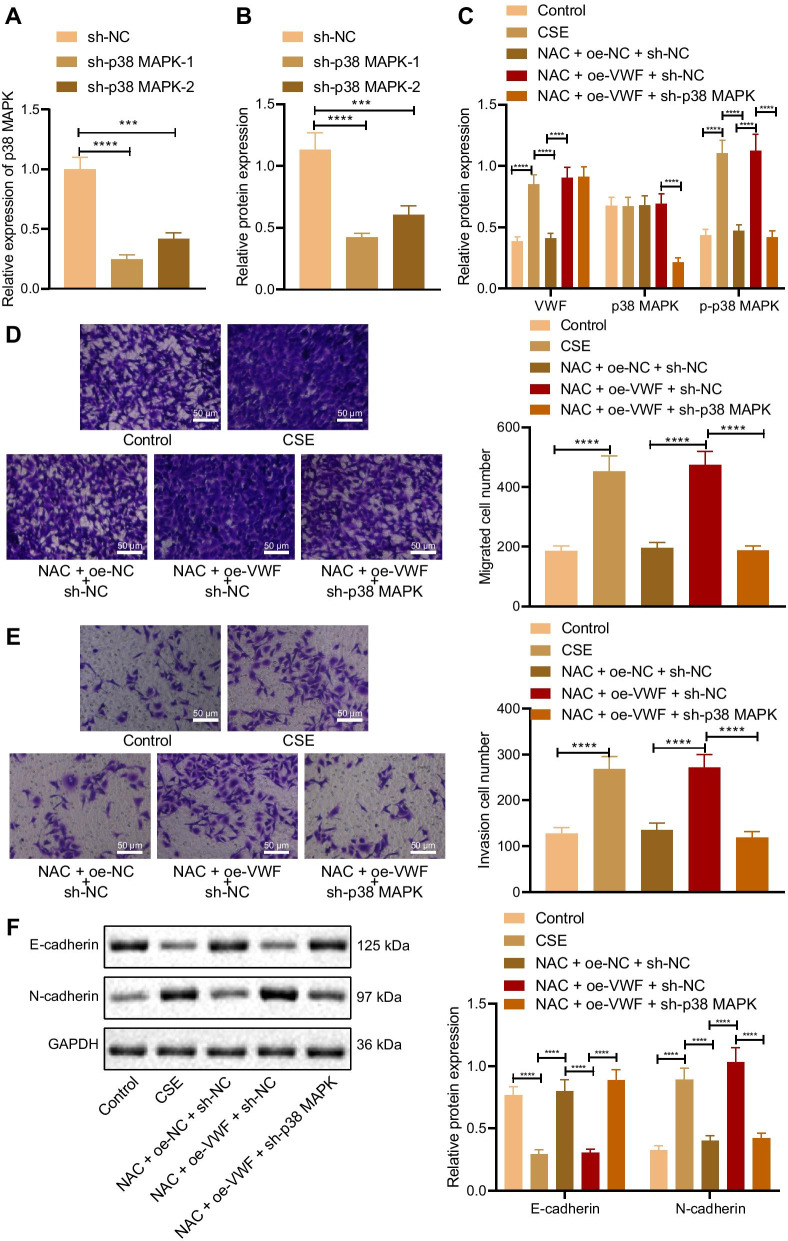
NAC affects EMT via the VWF/p38 MAPK axis. **A** Efficiency of sh-p38 MAPK-1 and sh-p38 MAPK-2 detected by RT-qPCR. **B** Efficiency of sh-p38 MAPK-1 and sh-p38 MAPK-2 detected by Western blot analysis. CSE cells were transduced with NAC and oe-VWF and/or sh-p38 MAPK. **C** Expression of VWF and p38 MAPK, and the extent of p38 MAPK phosphorylation in CSE cells determined by Western blot analysis. **D** CSE cell migration detected by Transwell assay. **E** CSE cell invasion detected by Transwell assay. **F** Protein levels of E-cadherin and N-cadherin in CSE cells determined by Western blot analysis. ***p < 0.001. ****p < 0.0001. Data are shown as the mean ± standard deviation of three technical replicates. Data among multiple groups were compared by one-way ANOVA with Tukey’s post hoc test

The effects of NAC inhibiting VWF/p38 MAPK on EMT of alveolar epithelial cells were further verified. RT-qPCR and Western blot analysis revealed that p38 MAPK expression reduced in cells transduced with sh-p38 MAPK-1 and sh-p38 MAPK-2, but this decline was more pronounced in cells transduced with sh-p38 MAPK-1. Thus, sh-p38 MAPK-1 (sh-p38 MAPK) was selected for the following experiments (Fig. 5A, B). RT-qPCR and Western blot analysis results presented that compared with cells without treatment, expression of VWF and the extent of p38 MAPK phosphorylation were increased, and p38 MAPK expression showed no significant difference in CSE cells. Compared with the CSE cells, expression of VWF and the extent of p38 MAPK phosphorylation were decreased, and p38 MAPK expression showed no significant difference in CSE cells transduced with NAC + oe-NC + sh-NC. Compared with the CSE cells transduced with NAC + oe-NC + sh-NC, expression of VWF and the extent of p38 MAPK phosphorylation were elevated, and p38 MAPK expression showed no evident difference in CSE cells transduced with NAC + oe-VWF + sh-NC. Compared with the CSE cells transduced with NAC + oe-VWF + sh-NC, expression of p38 MAPK and the extent of p38 MAPK phosphorylation were elevated, and VWF expression showed no obvious difference in CSE cells transduced with NAC + oe-VWF + sh-p38 MAPK (Fig. 5C). Transwell assay data displayed that, compared with cells without treatment, the CSE cell migration and invasion were promoted. Compared with the CSE cells, CSE cells transduced with NAC + oe-NC + sh-NC showed inhibited migration and invasion. Compared with the CSE cells transduced with NAC + oe-NC + sh-NC, cell migration and invasion were enhanced in CSE cells transduced with NAC + oe-VWF + sh-NC. Compared with the CSE cells transduced with NAC + oe-VWF + sh-NC, CSE cells transduced with NAC + oe-VWF + sh-p38 MAPK showed repressed cell migration and invasion (Fig. 5D, E). Western blot analysis exhibited that compared with cells without treatment, E-cadherin expression decreased and N-cadherin expression increased in CSE cells. Compared with the CSE cells, E-cadherin expression elevated and N-cadherin expression reduced in CSE cells transduced with NAC + oe-NC + sh-NC. Compared with the CSE cells transduced with NAC + oe-NC + sh-NC, E-cadherin expression decreased and N-cadherin expression increased in CSE cells transduced with NAC + oe-VWF + sh-NC. Compared with the CSE cells transduced with NAC + oe-VWF + sh-NC, E-cadherin expression elevated and N-cadherin expression reduced in CSE cells transduced with NAC + oe-VWF + sh-p38 MAPK (Fig. 5F).

### NAC promotes immune response in vivo by inhibiting VWF/p38 MAPK

**Fig. 6:**
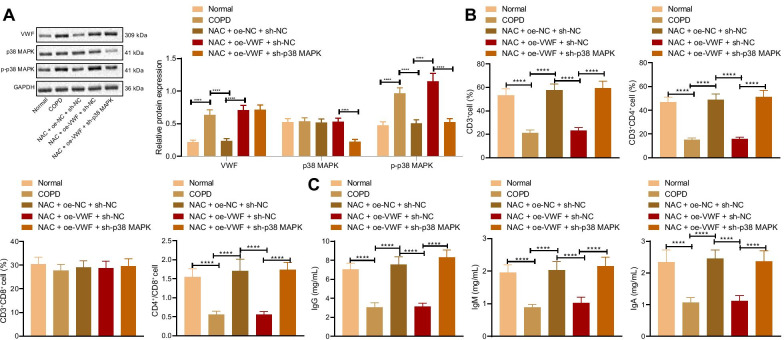
NAC affects the immune response in vivo through the VWF/p38 MAPK axis. COPD rats were treated with NAC and oe-VWF and/or sh-p38 MAPK (n = 10). **A** Expression of VWF and p38 MAPK, and the extent of p38 MAPK phosphorylation in lung tissues of COPD rats determined by Western blot analysis. **B** The ratio of CD3^+^, CD4^+^, CD8^+^ and CD4^+^/CD8^+^T lymphocytes in serum of COPD rats measured by flow cytometry. **C** Levels of IgG, IgA, and IgM in serum of COPD rats measured by ELISA. ****p < 0.0001. Data are shown as the mean ± standard deviation of three technical replicates. Data among multiple groups were compared by one-way ANOVA with Tukey’s post hoc test

Next, the effects of NAC promoting the immune response in vivo by inhibiting VWF/p38 MAPK axis were further explored. We injected the knockdown or overexpression vectors constructed into rats via tail vein to achieve the purpose of overexpression or knockdown of the target gene. Western blot analysis data presented that compared with the normal rats, the expression of VWF and the extent of p38 MAPK phosphorylation were increased, and p38 MAPK expression showed no significant difference in lung tissues of COPD rats. Compared with the COPD rats, expression of VWF and the extent of p38 MAPK phosphorylation were decreased, and p38 MAPK expression showed no significant difference in lung tissues of COPD rats treated with NAC + oe-NC + sh-NC. Compared with the COPD rats treated with NAC + oe-NC + sh-NC, expression of VWF and the extent of p38 MAPK phosphorylation were elevated, and p38 MAPK expression showed no evident difference in the lung tissues of COPD rats treated with NAC + oe-VWF + sh-NC. Compared with the COPD rats treated with NAC + oe-VWF + sh-NC, expression of p38 MAPK and the extent of p38 MAPK phosphorylation elevated, and VWF expression showed no obvious difference in lung tissues of COPD rats treated with NAC + oe-VWF + sh-p38 MAPK (Fig. 6A). Flow cytometry showed that compared with the normal rats, the ratio of CD3^+^, CD4^+^, CD8^+^ and CD4^+^/CD8^+^T lymphocytes decreased in serum of COPD rats. Compared with the COPD rats, the ratio of CD3^+^, CD4^+^, CD8^+^ and CD4^+^/CD8^+^T lymphocytes increased in serum of COPD rats treated with NAC + oe-NC + sh-NC. Compared with the COPD rats treated with NAC + oe-NC + sh-NC, the ratio of CD3^+^, CD4^+^, CD8^+^ and CD4^+^/CD8^+^T lymphocytes elevated in serum of COPD rats treated with NAC + oe-VWF + sh-NC. Compared with the COPD rats treated with NAC + oe-VWF + sh-NC, the ratio of CD3^+^, CD4^+^, CD8^+^ and CD4^+^/CD8^+^T lymphocytes reduced in serum of COPD rats treated with NAC + oe-VWF + sh-p38 MAPK (Fig. 6B). ELISA revealed that compared with the normal rats, levels of IgG, IgA, and IgM decreased in serum of COPD rats. Compared with the COPD rats, levels of IgG, IgA, and IgM increased in serum of COPD rats treated with NAC + oe-NC + sh-NC. Compared with the COPD rats treated with NAC + oe-NC + sh-NC, levels of IgG, IgA, and IgM elevated in serum of COPD rats treated with NAC + oe-VWF + sh-NC. Compared with the COPD rats treated with NAC + oe-VWF + sh-NC, levels of IgG, IgA, and IgM reduced in serum of COPD rats treated with NAC + oe-VWF + sh-p38 MAPK (Fig. 6C). These results indicated that NAC promoted the immune response in vivo by inhibiting VWF/p38 MAPK.

### NAC relieves COPD-induced pulmonary fibrosis by inhibiting VWF/p38 MAPK axis

**Fig. 7:**
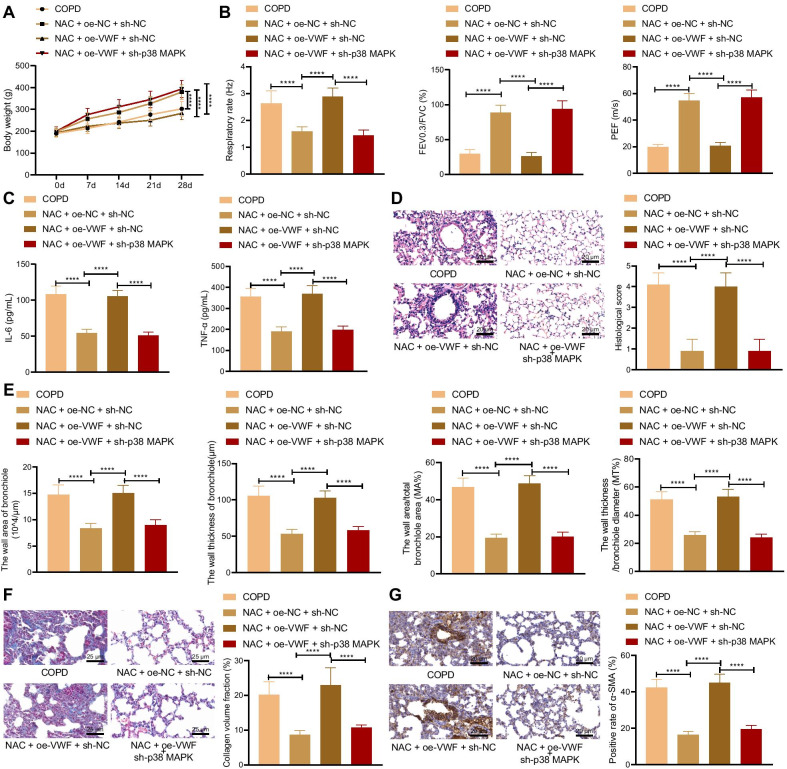
NAC relieves pulmonary fibrosis caused by COPD through the VWF/p38 MAPK axis. COPD rats were treated with NAC and oe-VWF and/or sh-p38 MAPK (n = 10). **A** The weight of COPD rats. **B** Pulmonary function of COPD rats. **C** Levels of IL-6 and TNF-α in the serum of COPD rats measured by ELISA. **D** Histological score of COPD rats detected by HE staining. **E** Bronchioles area, thickness bronchioles, the wall area/total bronchiole area (MA%) and the wall thickness/bronchiole diameter (MT%) of COPD rats. **F** Collagen volume fraction in lung tissues of COPD rats detected by Masson’s trichrome stain. **G** α-SMA level in lung tissues of COPD rats detected by Immunohistochemistry. Cells were treated with CSE and PBS or CSE and NAC. ****p < 0.0001. Data are shown as the mean ± standard deviation of three technical replicates. Data among multiple groups were compared by one-way ANOVA with Tukey’s post hoc test. Data at different time points were compared by two-way ANOVA with Bonferroni post hoc test

At last, whether NAC can alleviate COPD-induced pulmonary fibrosis by inhibiting the VWF/p38 MAPK axis was investigated. The results of the weight detection of rats showed that compared with the COPD rats, the weight of the COPD rats treated with NAC + oe-NC + sh-NC increased. Compared with the COPD rats treated with NAC + oe-NC + sh-NC, the weight of the COPD rats treated with NAC + oe-VWF + sh-NC reduced. Compared with the COPD rats treated with NAC + oe-VWF + sh-NC, the weight of the COPD rats treated with NAC + oe-VWF + sh-p38 MAPK increased (Fig. 7A). Evaluation of pulmonary function presented that compared with the COPD rats, respiration rate decreased, ratio of FEV_0.3_/FVC and PEF elevated in COPD rats treated with NAC + oe-NC + sh-NC. Compared with the COPD rats treated with NAC + oe-NC + sh-NC, respiration rate increased, ratio of FEV_0.3_/FVC and PEF reduced in COPD rats treated with NAC + oe-VWF + sh-NC. Compared with the COPD rats treated with NAC + oe-VWF + sh-NC, respiration rate decreased, ratio of FEV_0.3_/FVC and PEF increased in COPD rats treated with NAC + oe-VWF + sh-p38 MAPK (Fig. 7B). ELISA data displayed that compared with the COPD rats, levels of IL-6 and TNF-α decreased in the serum of COPD rats treated with NAC + oe-NC + sh-NC. Compared with the COPD rats treated with NAC + oe-NC + sh-NC, levels of IL-6 and TNF-α increased in serum of COPD rats treated with NAC + oe-VWF + sh-NC. Compared with the COPD rats treated with NAC + oe-VWF + sh-NC, levels of IL-6 and TNF-α elevated in serum of COPD rats treated with NAC + oe-VWF + sh-p38 MAPK (Fig. 7C). HE staining exhibited that compared with the COPD rats, COPD rats treated with NAC + oe-NC + sh-NC displayed less inflammation degree and more complete alveolar structure. Compared with the COPD rats treated with NAC + oe-NC + sh-NC, COPD rats treated with NAC + oe-VWF + sh-NC showed larger alveolar space and more infiltrating inflammatory cells. Compared with the COPD rats treated with NAC + oe-VWF + sh-NC, COPD rats treated with NAC + oe-VWF + sh-p38 MAPK exhibited reduced inflammatory cells and inflammation degree yet enlarged alveolar spaces (Fig. 7D). Compared with the COPD rats, the wall area, the wall thickness of the bronchioles, the wall area/total bronchiole area (MA%), and the wall thickness/bronchiole diameter (MT%) decreased in COPD rats treated with NAC + oe-NC + sh-NC. Compared with the COPD rats treated with NAC + oe-NC + sh-NC, the wall area, the wall thickness of the bronchioles, the wall area/total bronchiole area (MA%), and the wall thickness/bronchiole diameter (MT%) increased in COPD rats treated with NAC + oe-VWF + sh-NC (Fig. 7E). Masson’s trichrome stain and Immunohistochemistry presented that compared with COPD rats, collagen volume fraction and α-SMA level reduced in COPD rats treated with NAC + oe-NC + sh-NC, and compared with COPD rats treated with NAC + oe-NC + sh-NC, collagen volume fraction and α-SMA level increased in COPD rats treated with NAC + oe-VWF + sh-NC (Fig. 7F, G). The above results indicated that NAC alleviated pulmonary fibrosis caused by COPD by inhibiting VWF/p38 MAPK.

## Discussion

COPD is a progressive disease that eventually evolves into daily attacks, which seriously affects the quality of life of patients (Liu et al. 2016; Zheng et al. 2017). Although the clinical efficacy has been improved, COPD still has high incidence rate and mortality rate without obvious improvement (Su et al. 2020). In addition, patients suffering from COPD constitute a heterogeneous population with regard to treatment response (Carrasco Hernandez et al. 2021). It is of great significance to explore the pathological mechanism of COPD for finding effective therapeutic targets. Returning to the hypothesis in the beginning, we provided evidence in our study that NAC exerted inhibited properties in pulmonary fibrosis in COPD by promoting immune response and suppressing ETM through the downregulation of VWF/p38 MAPK axis.

Our initial observations revealed that COPD was accompanied with promoted cell migration and invasion, decreased E-cadherin expression, the ratio of CD3^+^, CD4^+^, CD8^+^ and CD4^+^/CD8^+^T lymphocytes, and levels of IgG, IgA, and IgM, and elevated N-cadherin expression and levels of IL-6 and TNF-α. An autoimmune component related to bronchial epithelial cell damage is possibly implicated in COPD progression and the presence of IgG and IgA is involved in COPD (Zhang et al. 2020). A recent study has also proved that ETM is a leading factor for COPD with decreased E‐cadherin expression and elevated levels of α‐SMA and collagen type I (Wang et al. 2019). The levels of proinflammatory cytokines, TNF-α and IL-1β are increased in the lungs of COPD patients and were suggested as potential targets (Pan et al. 2016). Mehani has confirmed that decreased the number of CD4 + cells and the ratio of CD4 + /CD8 + and upregulated IL-6 level are found in COPD (Mehani 2017). Moreover, the current study proved that NAC could promote the immune response and inhibit ETM to alleviate pulmonary fibrosis in COPD. The therapeutic effects of NAC on COPD have been investigated (Li et al. 2013). Due to its well-described antioxidant, anti-inflammatory, and mucolytic properties, NAC serves as a potential COPD therapy (Johnson et al. 2016). A recent study has revealed that NAC helps to prevent COPD exacerbation and improve pulmonary function (Shen et al. 2014). Its effects on COPD outcomes including exacerbation of and changes in lung function parameters are controversial (Fowdar et al. 2017). These findings support that NAC could ameliorate pulmonary fibrosis in COPD by facilitating immune response and repressing ETM.

In addition, the data in the present study also confirmed that VWF expression increased in COPD, and NAC could reduce the VWF expression to relieve pulmonary fibrosis in COPD. Similarly, NAC is reported to reduce the VWF in human plasma and mice (Chen et al. 2011). VWF acts as regulator in platelet activation and as a biomarker of endothelial dysfunction and inflammation in COPD (Polosa et al. 2013) Increased VWF levels could therefore potentially reflect the persistence of chronic inflammation in COPD (Langholm et al. 2020). Thus, the inhibition of VWF level has the potential to attenuate the pulmonary fibrosis in COPD. Furthermore, the obtained data suggested that NAC inhibited p38 MAPK phosphorylation by reducing the VWF expression to inhibit the pulmonary fibrosis in COPD. Apart from the antioxidant properties, NAC is known to suppress the activation of p38 MAPK pathway (Zhang et al. 2019). The activation of p38 MAPK is also found in COPD (Khorasani et al. 2015). The increased levels of MAPK phosphorylation in alveolar macrophages, bronchial epithelial cells, pulmonary lymphocytes and quadriceps femoris cells in patients with COPD suggest that the activation of MAPK pathway is involved in the pathogenesis of COPD (Lemire et al., 1985; Gaffey et al. 2013). p38 MAPK activation is also involved in local and systemic inflammation of COPD (Watz et al. 2014). Inhibition of p38 MAPK reduces the production of cytokines secreted by several lung and blood cells in COPD (Armstrong et al. 2011; Betts et al. 2015). These findings supported that NAC inhibited pulmonary fibrosis in COPD by inhibiting VWF/p38 MAPK axis.

## Conclusion

**Fig. 8:**
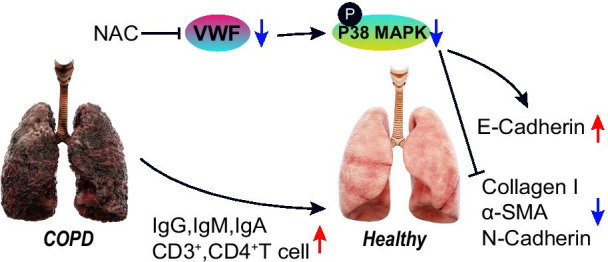
Antioxidant NAC promotes the immune response to improve COPD-induced pulmonary fibrosis by inhibiting the VWF/p38 MAPK/EMT axis

To sum up, our study demonstrated that NAC altered the VWF/p38 MAPK expression to enhance immune response and repress ETM, all of which leads to the inhibition of pulmonary fibrosis in COPD (Fig. 8). Our findings pave way for the development of effective therapeutic strategies for inhibiting pulmonary fibrosis in COPD. Due to the limited known researches, the roles of NAC, VWF, and p38 MAPK as well as their interaction in the pulmonary fibrosis in the progression of COPD should be more clearly investigated.

## Supplementary Information

**Additional file 1: Table S1.** Clinical and pulmonary function characteristics of all subjects. **Table S2** Silent sequence**. Table S3** Primer sequences for RT-qPCR.

**Additional file 2: Fig. S1.** Effect of VWF and NAC on the pathological degree following COPD. A, The expression of VWF determined by RT-qPCR in the lung tissues of COPD treated with NAC, sh-VWF or both. B, HE staining analysis of the pathological degree in the lung tissue of COPD treated with NAC, sh-VWF or both. C, Quantitative analysis of panel B. Data among multiple groups were analyzed by the one-way ANOVA with Tukey’s post hoc test. **** *p* < 0.0001.

**Additional file 3: Fig. S2.** Effect of VWF on the phosphorylation of p38 MAPK. A, Western blot analysis of VWF, p38-MAPK and p-p38-MAPK in cells treated with sh-VWF or oe-VWF. B, Quantitative analysis of panel A. Data among multiple groups were analyzed by one-way ANOVA with Tukey’s post hoc test. **** *p* < 0.0001.

## Data Availability

The datasets generated/analyzed during the current study are available.

## Abbreviations

CSE: Cigarette smoke extract
TNF-α: Tumor necrosis factor-α
EMT: Epithelial-mesenchymal transformation
COPD: Chronic obstructive pulmonary disease
CS: Cigarette smoke
NAC: *N*-Acetylcysteine
VWF: Von Willebrand factor
MAPK: Mitogen-activated protein kinase
FEV1: Forced expiratory volume in 1
FVC: Forced vital capacity
PMSF: Phenylmethylsulphonyl fluoride
ECL: Enhanced chemiluminescence
SABC: Streptavidin biotin peroxidase complex
ACK: Ammonium chloride-potassium
FITC: Fluorescein isothiocyanate
NC: Negative control
DMEM: Dulbecco's modified Eagle's medium
oe: Overexpression
sh: Short hairpin RNA
SD: Sprague–Dawley
BCA: Bicinchoninic acid
PEF: Peak expiratory flow
HE: Hematoxylin–eosin
RT-qPCR: Reverse transcription quantitative polymerase chain reaction
GAPDH: Glyceraldehyde-3-phosphate dehydrogenase
RIPA: Radio-immunoprecipitation assay
SDS-PAGE: Sodium dodecyl sulfate–polyacrylamide gel electrophoresis
PVDF: Polyvinylidene fluoride
ANOVA: Analysis of variance
