# Supplementary material for: The antioxidant N-acetylcysteine promotes immune response and inhibits epithelial-mesenchymal transition to alleviate pulmonary fibrosis in chronic obstructive pulmonary disease by suppressing the VWF/p38 MAPK axis
Source: Mol Med. 2021 Sep 3;27:97. doi: 10.1186/s10020-021-00342-y (PMC8414683; doi:10.1186/s10020-021-00342-y)
Supplement: Supplementary file 1 — Additional file 1: Table S1. Clinical and pulmonary function characteristics of all subjects. Table S2 Silent sequence. Table S3 Primer sequences for RT-qPCR. [file 10020_2021_342_MOESM1_ESM.docx]

**Table S1** Clinical and pulmonary function characteristics of all subjects

|  | Non-smokers (healthy) | Smokers with COPD |
| --- | --- | --- |
| Subject (n) | 10 | 10 |
| Age (years) | 60.8 ± 4.2 | 64.5 ± 3.8 |
| Sex (male/female) | 7/3 | 9/1 |
| Smoking history (pack-years) | 0 | 61.5 ± 5.2 |
| Smoking years | 0 | 46.6  ±  2.8* |
| FEV1 (% predicted) | 86.5 ±  4.4 | 32.8 ±  3.9* |
| FEV1/FVC (%) | 81.7  ±  6.2 | 47.1  ±  6.6* |
| GOLD stage |  |  |
| 1 | - | 1 |
| 2 | - | 7 |
| 3 | - | 2 |
| 4 | - | 0 |

Note: * *p* < 0.05 *vs.* non-smokers. COPD, chronic obstructive pulmonary disease.

**Table S2** Silent sequence

| Primers | Target sequence (5’-3’) |
| --- | --- |
| sh-NC | CCTAAGGTTAAGTCGCCCTCG |
| sh-p38 MAPK-1 | GGACCTCCTTATAGACGAATG |
| sh-p38 MAPK-2 | GGCACACTGATGACGAAATGA |

Note: NC, negative control; p38 MAPK, p38 mitogen-activated protein kinase.

**Table S3** Primer sequences for RT-qPCR

| Genes | Primer sequences (5’-3’) |
| --- | --- |
| VWF | F: GACTTCAAAGCCCCTGGACA |
|  | R: CCATACAAACAGGGGCCGTA |
| p38 MAPK | F: ATAATGCGTCTGACGGGGAC |
|  | R: GGGTCGTGGTACTGAGCAAA |
| GAPDH | F: AGTGCCAGCCTCGTCTCATA |
|  | R: ACCAGCTTCCCATTCTCAGC |

Note: RT-qPCR, reverse transcription quantitative polymerase chain reaction; VWF, von Willebrand factor; p38 MAPK, p38 mitogen-activated protein kinase; GAPDH, glyceraldehyde-3-phosphate dehydrogenase.
